# USDA Stakeholder Workshop on Animal Bioinformatics: Summary and Recommendations

**DOI:** 10.1002/cfg.266

**Published:** 2003-04

**Authors:** Debora L. Hamernik, David L. Adelson

**Affiliations:** 1 USDA-CSREES: (First Class Mail) 1400 Independence Ave SW; Stop 2220 Washington DC 20250-2220 USA; 2 USDA-CSREES: (Overnight Mail) Room 3441 Waterfront Centre 800 9th St. SW Washington DC 20024 USA; 3 Department of Animal Science Texas A&M University College Station TX 77843-2471 USA

## Abstract

An electronic workshop was conducted on 4 November–13 December 2002 to discuss current issues and needs in animal bioinformatics. The electronic (e-mail listserver) format was chosen to provide a relatively speedy process that is broad in scope, cost-efficient and easily accessible to all participants. Approximately 40 panelists with diverse species and discipline expertise communicated through the panel e-mail listserver. The panel included scientists from academia, industry and government, in the USA, Australia and the UK. A second ‘stakeholder’ e-mail listserver was used to obtain input from a broad audience with general interests in animal genomics. The objectives of the electronic workshop were: (a) to define priorities for animal genome database development; and (b) to recommend ways in which the USDA could provide leadership in the area of animal genome database development. E-mail messages from panelists and stakeholders are archived at http://genome.cvm.umn.edu/bioinfo/. Priorities defined for animal genome database development included: (a) data repository; (b) tools for genome analysis; (c) annotation; (d) practical application of genomic data; and (e) a biological framework for DNA sequence. A stable source of funding, such as the USDA Agricultural Research Service (ARS), was recommended to support maintenance of data repositories and data curation. Continued support for competitive grants programs within the USDA Cooperative State Research, Education and Extension Service (CSREES) was recommended for tool development and hypothesis-driven research projects in genome analysis. Additional stakeholder input will be required to continuously refine priorities and maximize the use of limited resources for animal bioinformatics within the USDA.


**Comparative and Functional Genomics**
*Comp Funct Genom* 2003; **4**: 271–274.
Published online 1 April 2003 in Wiley InterScience (www.interscience.wiley.com). **DOI:** 10.1002/cfg.266

**Conference Review**

### USDA Stakeholder Workshop on Animal Bioinformatics: summary and recommendations

Debora L. Hamernik[1]* and David L. Adelson[2]
[1] *USDA-CSREES, Washington, DC 20250-2220, USA*
[2] *Department of Animal Science, Texas A&M University, College Station, TX 77843-2471, USA*

**Correspondence to: Debora L. Hamernik, USDA-CSREES: (First Class Mail) 1400 Independence Ave, SW; Stop 2220, Washington, DC 20250-2220, USA; (Overnight Mail) Room 3441, Waterfront Centre, 800 9th St. SW, Washington, DC 20024, USA. E-mail: dhamernik@reeusda.gov*

Received: 29 January 2003
Revised: 31 January 2003
Accepted: 31 January 2003

### Abstract

**An electronic workshop was conducted on 4 November–13 December 2002 to discuss current issues and needs in animal bioinformatics. The electronic (e-mail listserver) format was chosen to provide a relatively speedy process that is broad in scope, cost-efficient and easily accessible to all participants. Approximately 40 panelists with diverse species and discipline expertise communicated through the panel e-mail listserver. The panel included scientists from academia, industry and government, in the USA, Australia and the UK. A second 'stakeholder' e-mail listserver was used to obtain input from a broad audience with general interests in animal genomics. The objectives of the electronic workshop were: (a) to define priorities for animal genome database development; and (b) to recommend ways in which the USDA could provide leadership in the area of animal genome database development. E-mail messages from panelists and stakeholders are archived at http://genome.cvm.umn.edu/bioinfo/. Priorities defined for animal genome database development included: (a) data repository; (b) tools for genome analysis; (c) annotation; (d) practical application of genomic data; and (e) a biological framework for DNA sequence. A stable source of funding, such as the USDA Agricultural Research Service (ARS), was recommended to support maintenance of data repositories and data curation. Continued support for competitive grants programs within the USDA Cooperative State Research, Education and Extension Service (CSREES) was recommended for tool development and hypothesis-driven research projects in genome analysis. Additional stakeholder input will be required to continuously refine priorities and maximize the use of limited resources for animal bioinformatics within the USDA.** **Copyright © 2003 John Wiley & Sons, Ltd.**

**Keywords:** **animals; bioinformatics; database; genomics**

### Introduction

Partial or complete DNA sequencing of domesticated farm animals is currently in progress for chickens and is planned for cattle and pigs in the near future. Genome sequence information has the potential for advancing knowledge relevant to human and animal health, improving animal production systems and adding value to animal products. In addition to benefits to agricultural production, animal genome sequence will be important for understanding the evolutionary relationships between species and to human health.

The availability of large amounts of genomic sequence will require that livestock genome researchers integrate sequence data, not only with existing gene maps but, more importantly, with QTL and phenotype data. Without integration, application of these data to agricultural enterprise productivity will be slow and inefficient. Thus, there is a clear need for additional bioinformatics resources,

Copyright © 2003 John Wiley & Sons, Ltd.

in the form of both databases and analytical tools aimed at increasing agricultural productivity and profitability.

The mission of the USDA includes improving animal production systems in the USA. Thus, it is likely that the USDA will continue to play a significant role in current and future research with animal genomics. The Agricultural Research Service (ARS) is the intramural research unit within USDA. ARS has a stable infrastructure and the ability to assign scientists to long-term and/or high-risk projects. The Cooperative State Research, Education and Extension Service (CSREES) is the extramural funding unit within the USDA. CSREES offers a variety of competitive grants programs to support basic and applied research projects. CSREES also supports the national Animal Genome Research Program (NRSP-8), which coordinates US genome efforts for cattle, sheep, swine, poultry, horses and aquatic species. Prioritization of programs within the USDA involves input from various stakeholders (scientists, commodity groups, etc.), advisory boards, strategic planning documents and Congressional mandates. To assist the USDA in setting priorities for animal bioinformatics, an electronic workshop was organized and conducted. The specific objectives of the electronic workshop were: (a) to define priorities for animal genome database development; and (b) recommend ways in which the USDA could provide leadership in the area of animal genome database development.

### Format of the electronic workshop

Two e-mail list servers were established to facilitate discussion and obtain input from (a) an invited panel of scientists interested in animal bioinformatics (i.e. the panel listserver) and (b) various stakeholders, including other researchers, professional organizations, commodity partners, etc. (i.e. the stakeholder listserver) with general interests in animal genomics. The electronic format was chosen to provide a relatively speedy process that was broad in scope, cost-efficient and easily accessible to all participants.

Invitations to participate in the electronic panel were distributed to approximately 50 scientists in US academic, government or private sector laboratories and to scientists in Australia and the UK. Approximately 40 scientists agreed to participate in the electronic workshop. The list of panelists is available at **http://genome.cvm.umn.edu/bioinfo/**

Comments submitted by panelists were not edited or filtered and were distributed simultaneously to all members of the panel. Although only the panelists could submit comments to the panel listserver, the panelists were strongly encouraged to consult broadly with colleagues and commodity partners who were not members of the panel and to share this input with the panel. Panelists were asked to check their e-mail messages at least once per day.

The stakeholder listserver was designed to capture additional input that would further broaden the panel's perspective in animal genomics. Invitations to participate in the electronic workshop through the stakeholder listserver were distributed to various species-specific listservers, including AnGenMap, LEPGEN-L, etc. Comments from the panel listserver and stakeholder listserver are also archived at **http://genome.cvm.umn.edu/bioinfo/**

Additional stakeholder input was obtained at the Animal Comparative Mapping Workshop at the Plant and Animal Genome (PAG) XI meetings in San Diego on 14 January 2002. A summary of all comments and recommendations is provided below.

### Comments and recommendations

Issues discussed in the electronic workshop and at the PAGXI meeting included identification of current and long-term needs and how these may be filled; discussion of ways to ensure interoperability, flexibility, curation and long-term support of animal genome databases; and development of strategies that could be used to prioritize and coordinate future national and international efforts for animal genome database development.

### Priorities for animal genome database development

To take advantage of genomic information, current, publicly available and permanent databases for animal genotypes and phenotypes are needed. In addition, web-based tools for querying these

Copyright © 2003 John Wiley & Sons, Ltd.

databases and data visualization to provide biological insight (rather than a list of things that are similar, based on DNA sequence) are needed. A recurring theme throughout the electronic discussion was, 'don't reinvent the wheel'. Another consistent recommendation was to focus on what needs to be done and not what is currently being done by other groups of investigators. The results of the electronic panel discussion identified the following priorities for animal genome database development: (a) a stable, comprehensive data repository; (b) tools for genome analysis; (c) annotation; (d) practical application of genomic data; and (e) a biological framework.

### Data repository

The National Center for Biotechnology Information (NCBI) has extensive experience with genome databases for a variety of species (**http://www.ncbi.nlm.nih.gov**). The resources at the NCBI are easily accessible by the public and relatively easy to use. The animal community should continue to take advantage of this resource and not 'reinvent the wheel' for data warehousing; thus, the NCBI may be an excellent location for a central data repository. The use of the NCBI as a data repository for animal genomes may also facilitate comparative genomics efforts. However, the NCBI may not be the only place where the primary sequence data may reside. This is because, among other things, QTL/linkage databases will have to include the genomic sequence of the QTL region, SNPs, STR polymorphism, and their relationships to phenotype.

### Tools for genome analysis

There is a clear need for development of tools to analyse DNA structure and function in animals. These tools should be publicly accessible and easy for everyone to use. Orthologue prediction may not be feasible, but clustering by protein coding sequence similarity across species to generate gene families is an initial step. Inclusion of QTL data is important and more complicated with animals, due to species and phenotypic differences. A 'tool' could in fact be a database (e.g. COG). It is likely that a separate QTL database will be required for each species. Comprehensive and up-to-date comparative mapping sites are also needed. Tools for mining heterogeneous sets of animal data (sequence, microarrays, proteomics, etc.) will be essential. Development of tools will most likely be done outside of NCBI, due to the need for high-level annotation of animal genomes and the need to emphasize specific aspects of livestock biology such as ruminant nutrition. Coordinated efforts with the NCBI will be needed to ensure optimum connectivity to other species databases. A mechanism or committee to evaluate, reinvent or remove tools from the system is also needed.

There is an immediate need to bring data from the agricultural community into the information management systems that already exist (e.g. PubMed). While agricultural journals are starting to be indexed by PubMed and PubMed Central, this process should be accelerated. Books of agricultural relevance should also be identified and added to the NCBI bookshelf. Alternatively, the National Agricultural Library (NAL) should serve the needs of the agricultural community in a similar manner that the National Library of Medicine (NLM) serves the medical community. USDA should plan to provide some funding to accelerate this process.

### Annotation

Annotation includes identification of all genes and determination of gene function as it relates to physiology and phenotypes that are recorded for each species. Annotation is considered to be an immediate priority for animal bioinformatics. Who will annotate the genome databases? A distributed annotation system may be necessary for the relatively small and scattered community of animal genome investigators that are located throughout the world. While an opportunity should be created for anyone to annotate databases, if there is no financial compensation for annotation it would not be expected that many investigators will spend a significant amount of time working on annotation. An annotation curator will likely be needed for accurate and consistent annotation.

### Practical application of genomic data

A vertebrate gene family database that can provide minimal identification of orthologues is considered a priority. Tools to identify orthologues, rapidly diverging orthologues and novel genes are also needed. The ability to update this database on a

Copyright © 2003 John Wiley & Sons, Ltd. 

frequent basis will be required. Since orthologue prediction may not be possible in many instances, gene families should be part of the biological database. A good example of a biological database is Online Mendelian Inheritance in Man (OMIM). These databases should include QTL data and be based on animal phenotypes/physiology.

### Biological framework

Above all, bioinformatics should be driven by 'biology' rather than by sophisticated analytical tools. The end result must be useful to physiologists and animal breeders, who may not know how to use complicated computerized tools. The sequence database should also include cellular, physiological (nutrition, stress, disease, etc.), and phenotypic data.

### Recommendations to the USDA for providing leadership in animal bioinformatics

At least two different mechanisms of funding will be needed to support the activities discussed above. First, a stable infrastructure is needed to support the maintenance of data repositories and curation of data. The stable source of funding is largely needed to support technical personnel rather than laboratory scientists. This source of stable funding will not likely come from competitive grants programs within the USDA–CSREES. Instead, database curation may be handled more effectively by the stable funding source within the USDA–ARS. Alternatively, the infrastructure within the NAL or the NCBI could be used for animal genome databases and partially supported by the USDA. In general, the NAL does not have personnel with the expertise to maintain and curate large databases; thus, the NAL was not considered to be a good choice for warehousing databases. Instead, a recommendation was made that the USDA should consider partnering with the NCBI to maintain and curate the animal genome databases. The NCBI is extremely interested in working with the animal genome community and, if financial support is available to hire additional personnel, the NCBI is willing to participate in more animal genome projects.

The second source of funding involves competitive grants programs within the USDA–CSREES. A recommendation was made that competitive grants should continue to be offered within USDA–CSREES to support tool development and hypothesis-driven research projects for genome analysis and application. Opportunities for funding bioinformatics tools should continue to be offered in the Animal Genome Basic Reagents and Tools Program within the National Research Initiative Competitive Grants Program. The USDA should work with the NCBI to identify gaps and develop short-term competitive grants programs to fill these needs.

In summary, this electronic workshop provided the first public opportunity for the US and international scientific communities to provide broad input to the USDA regarding current issues, needs and priorities in animal bioinformatics. The electronic workshop also facilitated coordination of national and international efforts in animal genome database development. The results from this workshop will be considered by the national program staff within USDA–CSREES when developing requests for applications (RFAs) related to animal genomics. Setting priorities for USDA competitive grants programs is a continuous process and will be influenced by future Congressional mandates, additional stakeholder workshops, advisory boards, scientific strategic planning documents, etc.

### Acknowledgements

The authors gratefully acknowledge Dr Vivek Kapur (University of Minnesota) for providing and maintaining the website and listserver for the electronic workshop. Gratitude is also expressed to Drs Ernie Bailey, Hans Cheng, Noelle Cockett, Jerry Dodgson, Max Rothschild and Jim Womack for identifying potential panelists. In addition, the authors extend sincere appreciation to the panelists and scientists attending the Animal Comparative Mapping Session at PAG XI for their insightful comments and suggestions.

Copyright © 2003 John Wiley & Sons, Ltd.

